# Structural Optimization and Interaction Study of a DNA Aptamer to L1 Cell Adhesion Molecule

**DOI:** 10.3390/ijms24108612

**Published:** 2023-05-11

**Authors:** Zhenhao Long, Tao Bing, Xiangru Zhang, Jing Sheng, Shuang Zu, Weiwei Li, Xiangjun Liu, Nan Zhang, Dihua Shangguan

**Affiliations:** 1 Beijing National Laboratory for Molecular Sciences, Key Laboratory of Analytical Chemistry for Living Biosystems, CAS Research and Education Center for Excellence in Molecular Sciences, Institute of Chemistry, Chinese Academy of Sciences, Beijing 100190, China; longzhuo@iccas.ac.cn (Z.L.); bingtao@iccas.ac.cn (T.B.); xiangru@iccas.ac.cn (X.Z.); shengjing@iccas.ac.cn (J.S.); zushuang21@mails.ucas.ac.cn (S.Z.); xjliu@iccas.ac.cn (X.L.); 2 School of Chemical Sciences, University of Chinese Academy of Sciences, Beijing 100049, China; 3 Zhejiang Cancer Hospital, The Key Laboratory of Zhejiang Province for Aptamers and Theranostics, Hangzhou Institute of Medicine (HIM), Chinese Academy of Sciences, Hangzhou 310022, China; 4 School of Molecular Medicine, Hangzhou Institute for Advanced Study, University of Chinese Academy of Sciences, Hangzhou 310013, China

**Keywords:** aptamer, L1CAM, sequence optimization, binding mechanism, molecular recognition

## Abstract

The L1 cell adhesion molecule (L1CAM) plays important roles in the development and plasticity of the nervous system as well as in tumor formation, progression, and metastasis. New ligands are necessary tools for biomedical research and the detection of L1CAM. Here, DNA aptamer yly12 against L1CAM was optimized to have much stronger binding affinity (10–24 fold) at room temperature and 37 °C via sequence mutation and extension. This interaction study revealed that the optimized aptamers (yly20 and yly21) adopted a hairpin structure containing two loops and two stems. The key nucleotides for aptamer binding mainly located in loop I and its adjacent area. Stem I mainly played the role of stabilizing the binding structure. The yly-series aptamers were demonstrated to bind the Ig6 domain of L1CAM. This study reveals a detailed molecular mechanism for the interaction between yly-series aptamers and L1CAM and provides guidance for drug development and detection probe design against L1CAM.

## 1. Introduction

The L1 cell adhesion molecule (L1CAM, also known as CD171) is a multi-structural domain type I transmembrane glycoprotein with a molecular weight of 200–220 kDa. It is a member of the immunoglobulin (Ig) superfamily and consists of six Ig-like domains followed by five fibronectin type III repeats, a transmembrane region, and a highly conserved intracellular domain [1]. L1CAM is mostly known for its important role in the development and plasticity of the nervous system, including neuronal migration and differentiation, neurite outgrowth, axon guidance, axon and dendrite fasciculation, synaptogenesis, and myelination [2]. Its variants can cause a variety of neurological diseases [3]. In addition, L1CAM has been reported to be highly expressed in a variety of tumors and plays a driving role in tumor formation, tumor progression, metastasis, invasion, and angiogenesis, making it a very promising target for tumor diagnosis and therapy [4] as well as a prognostic marker of several tumor types [2,5]. Currently, antibodies are the main recognition molecules used for detection and targeted therapy toward L1CAM [4]. There is no doubt that the development of new L1CAM ligands will greatly promote the functional research of L1CAM and the development of diagnostic reagents and drugs targeting L1CAM.

Aptamers are single-stranded oligonucleotides which can be selected and identified by the SELEX (Systematic Evolution of Ligands by EXponential enrichment) technique [6,7,8,9]. Aptamers can recognize and bind their target molecules with high affinity and specificity by forming thermodynamically stable three-dimensional structures [10], with the assistance of intramolecular non-covalent interactions. Lots of aptamers have been identified by various SELEX methods against different types of targets such as ions [11], organic small molecules [12], proteins [13], viruses [14], cells [15], and even tissues [16]. Many of them have shown excellent application prospects in the fields of affinity separation, biosensors, drug discovery, and diagnosis [17,18]. The cell-SELEX technique [15,19] (SELEX using cells as target) has shown great promise in generating cell-specific aptamers for discovery, detection, and cellular imaging of biomarkers [20] as well as for aptamer-based therapy [21,22,23]. However, among a large number of reported aptamers, only a few well-characterized aptamers have been widely used in scientific research and practical applications, such as aptamer sgc8 against PTK7 (protein-tyrosine kinase 7) [24], aptamer V7t1 against VEGF (vascular endothelial growth factor) [25], aptamer SL1 against c-MET (cellular-mesenchymal epithelial transition factor) [26,27], aptamer TBA against thrombin [28,29], and aptamer ABA against ATP [30]. This is because the original aptamers obtained by SELEX contain two fixed primer sequences, which are very long, complex in structure, low in yield, and poor in stability. Only after further characterization and optimization of the primary aptamers and understanding their interaction mechanism with the target molecule can they be widely used. The lack of effective means to investigate the real structure of aptamers and their interaction with target molecules seriously limits the further characterization of aptamers and hinders their wider application.

In our previous work, we identified a neurite-binding DNA aptamer, yly12, using a modified cell-SELEX strategy (neurite-SELEX). It was further identified to specifically bind L1CAM on the surface of neuron cells and some cancer cell lines. Yly12 was found to inhibit the growth of neurites and was successfully used for imaging the neurite network formed by cultured neuronal cells and nervous fiber in brain tissue sections [31]. Based on the important roles of L1CAM in the development of the nervous system and tumors, here, we further optimized aptamer yly12 and explored its interaction with L1CAM, hoping to provide a new tool for the functional research of L1CAM and provide new information for the design of diagnostic reagents and therapeutic drugs targeting L1CAM.

## 2. Results and Discussion

### 2.1. The Optimization of Aptamer yly12

In our previous study, aptamer yly12 was the sequence with the strongest affinity in a group of sequences truncated gradually from the 3’ or 5’ ends of the original aptamer ylQ3. The apparent dissociation constant (*K*_d_) of yly12 was measured to be 3.5 ± 2.4 nM at 4 °C [31]. Because many applications of this aptamer will be conducted at room temperature and physiological temperature, the affinities of yly12 at 25 °C and 37 °C were measured by flow cytometry using cells with highly expressed L1CAM. The *K*_d_ values of yly12 at 25 °C and 37 °C were 21.6 ± 2.0 nM and 110.8 ± 21.7 nM, respectively, which are 6 and 31 times higher than that at 4 °C, suggesting that its performance in practical applications would be greatly reduced. In order to obtain an aptamer with stronger affinity and determine its key nucleotides for binding to L1CAM, aptamer yly12 was further optimized and mutated.

**Figure 1:**
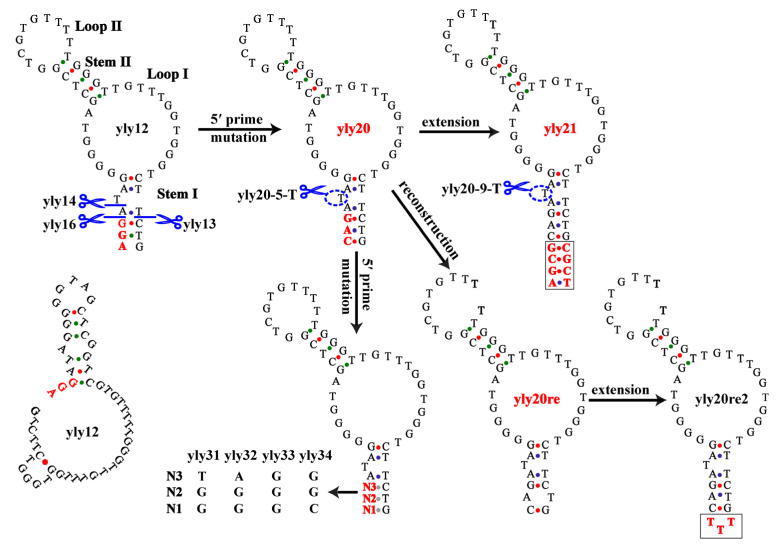
Predicted secondary structures of aptamer yly12 and its mutated sequences. Bold red letters indicate the changed nucleotides and, in particular, black squares indicate the extended nucleotides. Scissors indicate the site of truncation. Scissor + dashed ellipse indicate the removal of the nucleotide in dashed ellipse.

The secondary structures of yly12 were predicted using Mfold [32], and two possible structures are shown in Figure 1. Because the first predicted structure of yly12 (bottom left of Figure 1, also shown in our previous paper) does not conform to the secondary structure of most reported aptamers, the aptamer optimization was performed based on the predicted secondary structure. For convenience of description, the structure of yly12 was divided into four parts: Stem I, Stem II, Loop I and Loop II (Figure 1). Our previous work showed that further truncating three or four nucleotides from 5′ or 3′ end of yly12 (yly13, yly14, yly16; Figure 1 and Table 1) greatly decreased but did not completely eliminate its affinity [31], suggesting that stem I might not be the most critical region for binding to L1CAM, but that it might play a role in the stabilization of the binding structure. Figure 1 shows that the 5′-end nucleotides AG and 3′-end nucleotides GT in stem I cannot form fully complementary base pairs (they only form a wobble base pair G-T). In addition, there is a thymine bulge in the middle of stem I, which may lead to a decrease in the stability of the binding structure and therefore a decrease in the affinity of yly12 at 25 and 37 °C.

Based on the above speculation, aptamer yly20 was designed via mutating the 5′-end nucleotides AG of yly12 to CA in order to form two Watson–Crick base pairs at the terminal of stem I (Figure 1). The affinity of yly20 to L1CAM at 4 °C was slightly enhanced compared with yly12, with a *K*_d_ of 2.3 ± 0.4 nM, but the affinities at 25 °C and 37 °C were greatly enhanced with *K*_d_ values of 1.9 ± 0.3 nM and 7.6 ± 1.0 nM, respectively (Table 1). Further prolonging stem I of yly20 through adding three G-C base pairs and one A-T base pair at the terminal (aptamer yly21) led to a further increase in its affinities (*K*_d_ = 1.3 ± 0.4, 0.9 ± 0.3, and 5.1 ± 0.5 nM) at 4 °C, 25 °C, and 37 °C (Table 1). To further verify if stem I plays a critical role in maintaining the affinity of yly20, mutant sequences yly31, yly32, yly33, and yly34 were designed via replacing the 5′-end nucleotides CAG with GGT, GGA, GGG and CGG, respectively, to reduce the Watson–Crick base pairs in stem I (Figure 1). The *K*_d_ values of these aptamers under different temperatures were measured (Table 1) and showed that sequences with more mismatched base pairs have lower affinity. These results suggest that a stable stem I plays an important role in maintaining the aptamer-binding structure.

In order to further increase the stability of stem I, sequences yly20-5-T and yly21-9-T were designed via removing the single T bulge in stem I of yly20 and yly21, respectively. The *K*_d_ values of yly20-5-T and yly21-9-T at 4 °C unexpectedly increased to 112 ± 20 nM and 107 ± 28 nM, respectively (Figure 1 and Table 1), which were much higher than all the above mutated sequences. The greatly reduced affinities suggest that the single T bulge is critical for aptamer binding or maintaining the binding structure. In addition, two reconstructed aptamers (yly20re and yly20re2) were designed simply via splitting aptamer yly20 between the nucleotides T26 and T27 and connecting its original 3′ and 5′ ends (yly20re) or connecting its original 3′ and 5′ ends through TTT (yly20re2) (Figure 1). The *K*_d_ values showed that compared with yly20, the affinity of yly20re only decreased slightly at all tested temperatures, whereas the affinity of yly20re2 decreased slightly at 4 °C and decreased more at 25 °C and 37 °C. However, yly20re had better affinity than the original aptamer yly12 at all the tested temperatures (Table 1). This set of results indicates that stem I is necessary for maintaining the binding structure of yly-series aptamers (for example, maintaining the formation of loop I).

**Figure 2:**
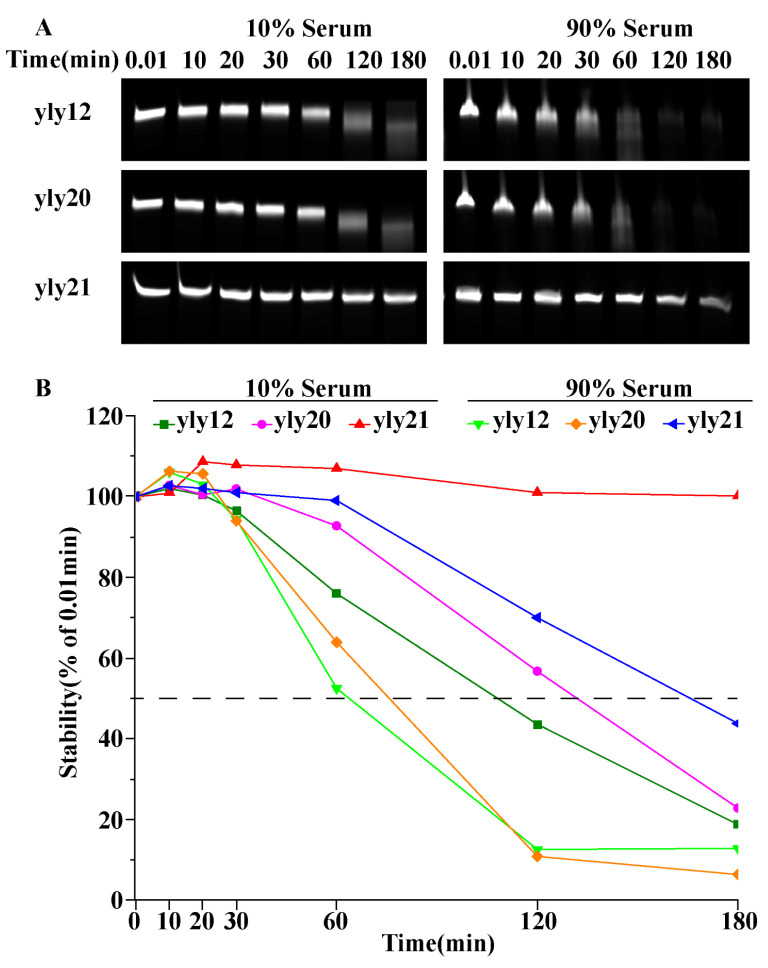
The polyacrylamide denaturing gel electrophoresis of aptamers (yly12, yly20 and yly21) after incubation in different concentrations of FBS at 37 °C for different times (**A**). (**B**) represents degradation curve based on grayscale values in (**A**).

For the application of these aptamers in diagnosis and therapy, their stability in biological fluids needed to be considered. Therefore, the stability of yly12, yly20, and yly21 was also tested in different concentrations of fetal bovine serum (FBS) (Figure 2). Compared to yly12, yly20 showed a slight improvement in its resistance to nuclease degradation in 10% and 90% FBS, but yly21 showed a significant improvement. Most of yly21 stayed intact for up to 3 h in 10% FBS, and 45% of yly21 stayed intact for up to 3 h in 90% FBS. This result suggests that besides higher affinity, the optimized aptamers also have better biostability in FBS. Combined with other chemical modifications, it is believed that sufficient stability of the optimized aptamers could be achieved for in vivo application.

### 2.2. Investigation into Binding Structure and Key Nucleotides of Aptamer yly20

The proportion of G nucleotides in the regions of loop I, stem II, and loop II are high (19/40), suggesting the possibility of forming a G-quadruplex structure. To further characterize the binding structure and the key nucleotides for aptamer binding, circular dichroism (CD) spectroscopy, CD-melting, methylation interference assay, and mutation experiments were performed. The CD spectra of sequences yly12, yly20, and yly21 did not show any characteristic signal of G-quadruplexes (Figure S1). Further, in order to reduce the effect of stem I on the G-quadruplex formation, yly20-7 was designed via removing seven nucleotides from the end of stem I of yly20. The CD spectrum of sequence yly20-7 did not show any G-quadruplex signal either, suggesting no G-quadruplex in the binding structure of yly20. In addition, the structural stability of yly12, yly20 and yly21 was investigated using a CD-melting experiment (Figure S1b). The *T_m_* values of yly12, yly20, and yly21 were 37 °C, 50 °C, and 54 °C, respectively, suggesting that the structural stability of these aptamers increased with the stability of their stem I, as the structures of yly20 and yly21 were much more stable than that of yly12. These results are consistent with the previous affinity measurement results.

**Figure 3:**
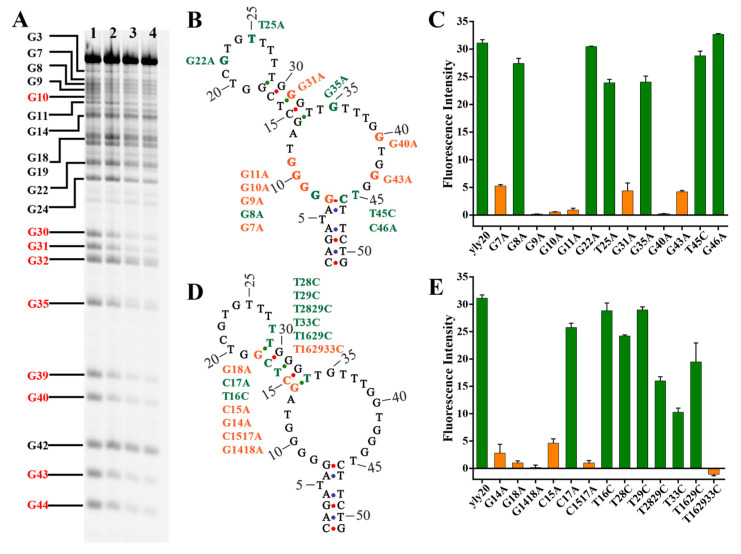
(**A**) DMS footprinting of 3′FAM-labeled yly20. Lane 1, yly20 in ddH_2_O; lane 2, yly20 in PBS; lanes 3–4, yly20 + L1CAM. The protected G nucleotides were highlighted in red. (**B**,**D**) Mutation site illustration of yly20 mutation sequences. Yellow colors indicate that mutated sequences did not bind or only weakly bound to L1CAM; Green letters indicate that the mutated sequences still strongly bound to L1CAM. (**C**,**E**) Binding of FAM-labeled mutated sequences of yly20 to LoVo cells. The meaning of colors are consistent with that in (**B**,**D**).

Methylation interference assay (also named dimethyl sulfate (DMS) footprinting) is widely used to investigate the interaction of DNA with proteins [33] as well as the structural change of DNA [34]. DMS can methylate the N7 position of the G nucleotides in a DNA sequence, and the methylated G residues can be, in turn, chemically cleaved by piperidine. If an aptamer forms a quadruplex, triplex, or tertiary structure, or if it binds to a protein, the N7 position of the G nucleotides that is blocked in the formation of the special structures or by the protein will be protected from DMS modification, resulting in the band of G methylation on the gel to weaken. As shown in Figure 3A, compared with the DMS reaction of yly20 in water (lane 1), the DMS reaction in PBS (lane 2) only showed a slight effect on protecting nucleotides G10, G30, G31, G39, G40, G43, and G44 from methylation. Because the binding structure of yly20 only formed in PBS and not in water, this result suggests that the slight protection of these G nucleotides may result from the formation of the binding structure, but not the formation of a G-quadruplex structure. The DMS reaction of yly20 in the presence of L1CAM (lane 3 and 4) showed a strong protective effect on nucleotides G10, G30, G31, G35, G39, G40, G43, and G44 from methylation, suggesting that these G nucleotides may be involved in the binding of yly20 to L1CAM.

To further understand the binding structure of yly20, the protected nucleotides G10, G31, G35, G40 and G43 found in above DMS experiment, and the nucleotides G7, G8, G9, G11, G22, C46 and T25 in loop I and loop II (Figure 3B) were mutated to nucleotide A respectively. The binding ability of the mutated sequences was measured through a flow cytometry experiment (Figure 3C). The mutated sequences yly20-G9A, yly20-G10A, yly20-G11A, and yly20-G40A almost lost their binding ability to L1CAM, while the sequences yly20-G7A, yly20-G31A, and yly20-G43A showed greatly reduced binding to L1CAM, suggesting that nucleotides G7, G9, G10, G11, G31, G40, and G43 play a key role in the direct binding to L1CAM or in the formation of the binding structure. However, the sequences yly20-G8A, yly20-G35A, and yly20-C46A, although still maintaining strong binding ability, all had increased *K*_d_s compared to yly20 (Table 2), suggesting that nucleotides G8, G35, and C46 are important but not critical for aptamer binding.

Interestingly, the mutation of G22 (yly20-G22A) and T25 (yly20-T25A) in loop II almost did not affect aptamer-binding ability, together with the slightly reduced binding ability of the reconstructed aptamers (yly20re and yly20re2, Figure 1) whose loop II was split between T26 and T27, which suggests that loop II might not take part in the binding of the aptamer to L1CAM.

It is worth noting that the mutation of G7 to A (yly20-G7A, decrease in base pairs of stem I) greatly reduced aptamer-binding ability, but the mutation of C46 to A (yly20-C46A, decrease in base pairs of stem I), G8 to A (yly20-G8A, increase in base pairs of stem I), and T45 to C (yly20-T45C, increase in base pairs of stem I) only partly reduced binding ability (Figure 3). These results, together with the above result that removal of the single T bulge (increase in the stability of stem I) led to greatly decreased binding ability, suggests that the formation of base pairs A6-T47 and G7-C46 may not be very important for aptamer binding, and that the nucleotide G7 may take part in directly binding to L1CAM. Because the two base pairs are directly adjacent to loop I, and a large part of the G nucleotides in loop I are critical for aptamer binding, loop I may be the direct binding region of the aptamer to L1CAM.

To investigate the role of stem II of yly20 in its interaction with L1CAM, a series of sequences was designed via mutating 1–3 nucleotides in stem II and loop II. As a general understanding, the mutation of nucleotides G14 and/or G18 to A will strengthen the stability of stem II because of the replacement of the wobble G-T base pair with the Watson–Crick A-T base pair; however, the mutated sequences yly20-G14A, yly20-G18A, and yly20-G1418A (both nucleotides mutated) almost lost their binding ability, suggesting that G14 and G18 may be the key nucleotides for binding (Figure 3D,E). The mutation of nucleotides T16, T28, T29, and T33 to C convert the wobble G-T base pair to the Watson–Crick G-C base pair. The single-nucleotide-mutated sequences yly20-T28C, yly20-T29C, and yly20-T33C retained considerable binding ability, but yly20-T16C lost some affinity. The two-nucleotides-mutated sequence yly20-T2829C lost some affinity and yly20-T1629C lost more affinity, but the three-nucleotides-mutated sequence yly20-T162933C almost totally lost its binding ability (Figure 3D,E and Table 2). Mutation of nucleotides C15 and C17 to A convert the Watson–Crick G-C base pair to a G-A mismatch in stem II. The mutated sequences yly20-C15A and yly20-C1517A significantly lost their affinity to L1CAM, but yly20-C17A maintained its binding ability. This set of results seems to be contradictory. Increasing the number of Watson–Crick base pairs in stem II did not affect the binding affinity of the aptamer, and decreasing the number of Watson–Crick base pairs in stem II also caused the same phenomenon. These results suggest that some nucleotides in stem II not only participate in the formation of stem II, but may also participate in direct binding with L1CAM, and that L1CAM binding may induce structural changes of yly20.

### 2.3. The Aptamer-Binding Domain of L1CAM

**Figure 4:**
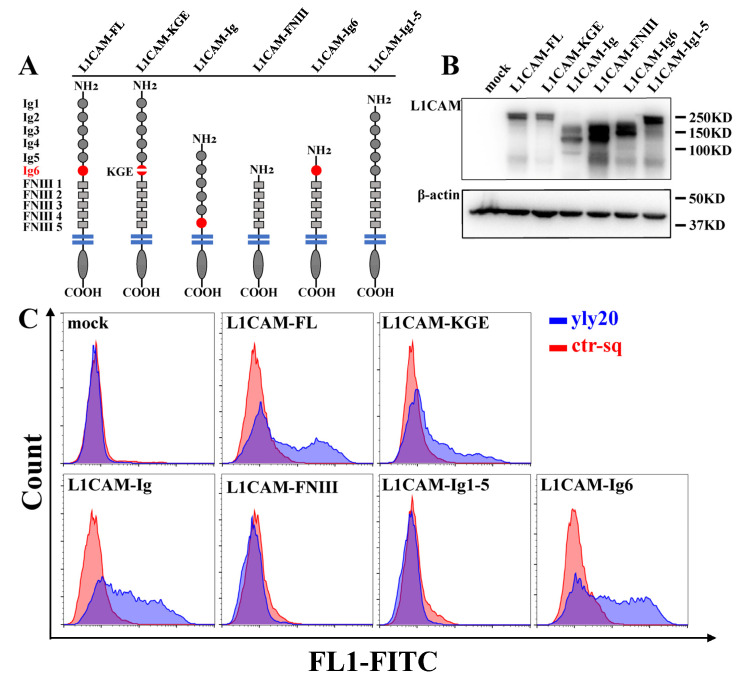
Binding of yly20 to L1CAM deletion mutants in overexpressed cells. (**A**) Graphical representation of L1CAM deletion mutants with different lengths. (**B**) Western blot analysis of the expression of L1CAM deletion mutants in PC3 cells transfected with plasmids with different lengths. (**C**) Binding of yly20 on PC3 cells transfected with different plasmids.

As a transmembrane protein, the extracellular domain of L1CAM consists of six immunoglobulin-like domains (Ig-domains) and five fibronectin type III (FNIII) repeats. These different domains interact with different binding partners—which can exist on other cells, extracellular matrices, or in extracellular fluid—such as integrins, activated leukocyte cell adhesion molecules, E-selectin, neuropilin 1, and FGFR, thus exercising different biological functions such as cell adhesion, differentiation, migration, proliferation, and invasion [1]. To determine which domain in L1CAM is important for binding to yly20, we overexpressed a series of L1CAM deletion mutants on L1CAM-negative cells and tested the aptamer binding. First, we constructed three L1CAM plasmids with different lengths: an L1CAM full-length plasmid (L1CAM-FL), an L1CAM-Ig plasmid with all the fibronectin type III repeats removed, and an L1CAM-FNIII plasmid with the immunoglobulin-like domains removed (Figure 4A). All three constructed plasmids and the control plasmid were respectively transfected into PC3 cells that did not express L1CAM. Western blot experiments confirmed that all plasmids were successfully expressed in PC3 cells (Figure 4B). The flow cytometry assay showed that yly20 bound PC3 cells transfected with the L1CAM-FL plasmid and L1CAM-Ig plasmid but did not bind PC3 cells transfected with the L1CAM-FNIII plasmid or the control plasmid (Figure 4C), suggesting that yly20 binds to the immunoglobulin-like domains of L1CAM protein.

Further, we constructed two additional plasmids, an L1CAM-Ig6 plasmid with the Ig1–Ig5 domains removed and an L1CAM-Ig1-5 plasmid with the Ig6 domain removed (Figure 4A). Both constructed plasmids and the control plasmid were transfected with PC3 cells. The flow cytometry assay showed that yly20 bound PC3 cells transfected with the L1CAM-Ig6 plasmid but did not bind PC3 cells transfected with the L1CAM-Ig1-5 plasmid or the control plasmid (Figure 4C), suggesting that yly20 binds to the Ig6 region in the immunoglobulin-like structural domain of L1CAM.

The Ig6 domain of L1CAM contains an Arg-Gly-Asp (RGD) motif that is the cell attachment site of adhesive extracellular matrices, blood, and cell surface proteins [1]. The RGD motif is mainly involved in the interaction with various integrins that serve as RGD receptors and constitutes one of the main recognition systems for cell adhesion. To clarify whether the RGD motif in the Ig6 region of L1CAM directly binds aptamer yly20, we constructed the plasmid L1CAM-KGE, with the mutation of arginine (R554) and aspartic acid (D556) in the RGD motif to lysine (K) and glutamic acid (E), and transfected it into PC3 cells. The flow cytometry assay revealed that yly20 bound to PC3 cells transfected with the L1CAM-KGE plasmid, suggesting that the RGD motif may not directly bind yly20. As an action site of integrin and integrin ligands, RGD sequences play an extremely important role in various cellular behaviors, such as adhesion, migration, infiltration, and proliferation. The synthesized RGD tripeptide and peptides containing the RGD motif alone can also inhibit the interaction between integrin and integrin ligands, and are widely used in biomedical research, tissue engineering, drug delivery, diagnosis, and therapy [35]. Thus, the effect of RGD peptides on the binding of yly20 to L1CAM was investigated through a competitive binding experiment using a synthesized RGD peptide (Arg-Gly-Asp) and an Ig6-25 peptide (aa544-568 in Ig6 of L1CAM, containing an RGD motif). Both the RGD peptide and Ig6-25 peptide could not compete for the binding of yly20 to L1CAM (Figure S2), suggesting that the binding of yly20 to L1CAM involves the entire structure of the Ig6 domain rather than some specific amino acid sequences.

To determine the binding site of the aptamer in L1CAM, we performed a chemical footprinting experiment via p-hydroxyphenyl glycol (HPG) modification of arginine. HPG is a protein modification reagent that reacts with arginine residues in proteins. The binding of ligands to a protein will protect the arginine residues from modification by HPG [36]. L1CAM was reacted with HPG after incubation with a control aptamer (Ctr-sq) and yly20, respectively. The samples were separated and purified using SDS-PAGE gel electrophoresis and subjected to in-gel digestion with trypsin. The HPG-labeled peptides were analyzed using LC-MS (Figure S3). Ten arginine sites modified by HPG were identified in the control group (Ctr-sq) (Figure S3 and Table S4) and four of them were protected from the HPG reaction by aptamer yly20, in which arginine residues R534 and R575 were in the Ig6 domain (aa518–607) of L1CAM, and arginine residue R617 was very close to the Ig6 domain. Another arginine residue R228 between the Ig2 and Ig3 domains was also protected, while arginine site R238 was overmodified in the presence of yly20. This result is consistent with the plasmid transfection experiments and the peptide competition experiments described above, demonstrating that yly20 binds to the Ig6 domain of the L1CAM protein.

### 2.4. Structural Prediction and Molecular Docking

**Figure 5:**
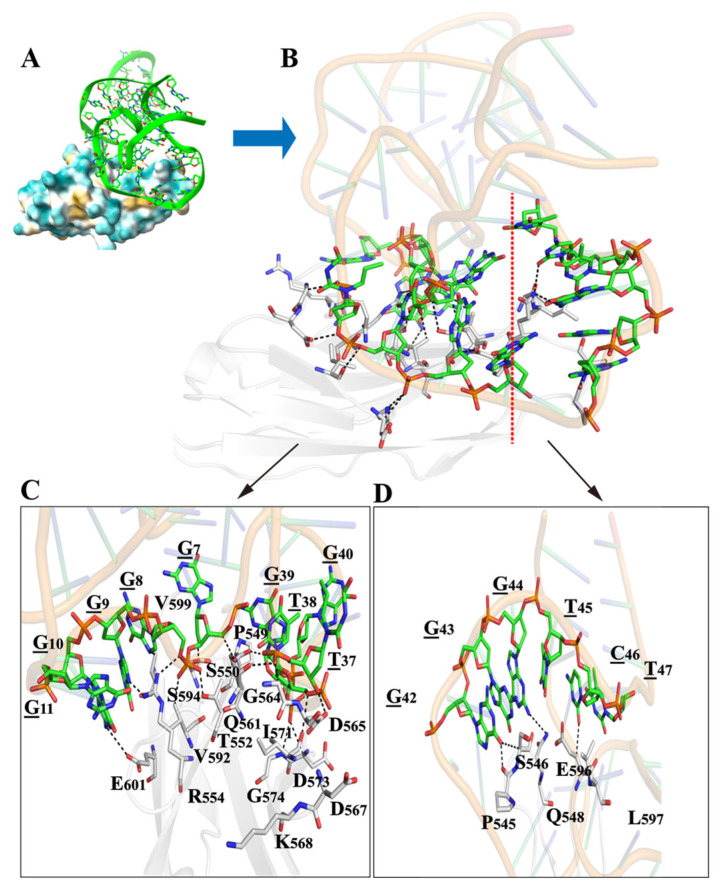
The molecular docking simulation of yly20 and Ig6 domain. (**A**) The best-performing docking conformation (model 9); the Ig6 domain is shown in different-color surfaces, and the phosphate backbone of yly20 is shown in green. (**B**–**D**) The overall and detailed views of interactions involving amino acids and bases. The phosphate backbone is shown as a thick orange circular line and the peptide chain is represented by a white cartoon diagram. Hydrogen bonding interactions are indicated by black dashed lines. The red dotted line in (**B**) represents the division of the complex structure into two parts, (**C**,**D**), indicated by the arrows, from here on.

In order to further understand the binding of yly20 to L1CAM, a molecular docking study was performed. The PDB database does not include any crystal or NMR data of L1CAM. The extracellular region of L1CAM is composed of six Ig-like domains and five fibronectin type III repeats, which are reported to adopt a dynamic, integrated structure with modular and cooperative interaction modes among the different domains [37]. The above results have demonstrated that aptamer yly20 binds the Ig6 domain; thus, the Ig6 domain was chosen for the docking study. The 3D structure of yly20 (Figure S4A) was constructed via 3dRNA/DNA [38] according to the above experimental results, and the secondary structure information was predicted using Mfold [32] (Figure 1). The 3D structure of the Ig6 domain was constructed using Alphafold from the Uniport database (Figure S4B). Docking was performed using the HDOCK molecular docking web server [39] and the top 10 yly20-Ig6 complexes with the highest docking scores are shown in Table S2. The free energy of docking of the best yly20-Ig6 complex was −182.12 kcal/mol, and its 3D structure and hydrogen bonds between amino acid and nucleotide residues are shown in Figure 5 and Table S3. For a clearer and more detailed presentation, the interaction interface was divided into two parts (Figure 5C,D). The interacting hydrogen bonds specifically involved amino acid sites P545, S546, Q548, P549, S550, T552, R554, Q561, G564, D565, I571, D573, G574, V592, S594, E596, V599, and E601, as well as nucleotide sites G7, G8, G9, G10, T37, T38, G39, G42, G44, and C46. The binding region of yly20 is mainly located in loop I and its adjacent area. The docking results were basically consistent with the previous experimental results. Compared with most proteins, the structure of this aptamer is relatively flexible; its structure may be induced to change when binding a target protein. Therefore, the results of the docking simulation may not be completely consistent with the real binding structure, and more experiments, such as NMR or single-particle cryo-electron microscopy, may reveal the real binding structure.

## 3. Materials and Methods

### 3.1. Reagents and Buffers

The L1CAM protein was purchased from Sino Biological Inc. (Beijing, China). All oligonucleotides (Table S1) were synthesized and HPLC-purified by Sangon Biotechnology Co. Ltd. (Shanghai, China). All reagents were purchased from Sigma Aldrich Chemical Co. (St. Louis, MO, USA) unless otherwise specified. Roswell Park Memorial Institute medium (RPMI1640), fetal bovine serum (FBS), and Penstrep (penicillin/streptomycin solution) were from Life Technologies (Shanghai, China). P-Hydroxyphenylglyoxal (HPG) was purchased from YuanYe Bio-Technology Co. Ltd. (Shanghai, China). The NCAM-L1 (C-2) mouse monoclonal antibody was purchased from Santa Cruz Biotechnology, Inc. (Dallas, TX, USA) RGD (Arg-Gly-Asp) peptide was purchased from Selleck. L1CAM-Ig6-25 peptide (DPSLQPSITWRGDGRDLQELGDSDK) was synthesized and purified by GL Biochem Ltd. (Shanghai, China). The washing buffer was supplemented with glucose (4.5 g/L) and MgCl_2_ (5 mM) in phosphate buffer saline (PBS). The binding buffer was supplemented with bovine serum albumin (BSA, 1 mg/mL) and herring sperm DNA (HS-DNA, 0.1 mg/mL) into the washing buffer. The oligonucleotide lyophilized powders were dissolved in PBS and the concentrations were determined based on UV absorption of 260 nm. Prior to use, all oligonucleotide solutions were denatured via heating at 95 °C for 5 min, followed by rapid cooling on ice for 10 min and then annealing at room temperature for 30 min. The denatured oligonucleotide solutions were stored at −20 °C.

### 3.2. Cell Lines and Cell Culture

LoVo (colon cancer) and PC3 (prostate cancer) cell lines were purchased from the Typical Culture Preservation Commission Cell Bank, Chinese Academy of Sciences (Shanghai, China), and were cultured in RPMI-1640, supplemented with 10% FBS and 1% Penstrep, in a humidified atmosphere with 5% CO_2_ at 37 °C.

### 3.3. Flow Cytometry Analysis

Flow cytometry assays were performed for the measurement of aptamer-cell-binding capacity and the acquisition of *K*_d_ values. Cells in the exponential growth phase were dissociated using a PBS solution containing 5 mM EDTA and blown up into monodisperse cell suspensions. After centrifugation and washing with a washing buffer, the cells were diluted and resuspended in binding buffer. For the cell binding assay, 200 nM FAM-labeled aptamers were incubated with cells for 30 min at 4 °C in a 100 µL system. For the aptamer *K*_d_-value determination, a series of concentration gradients of FAM-labeled aptamers (1.56, 3.12, 6.25, 12.5, 25.0, 50.0, 100, and 200 nM) were prepared and incubated with cells in a 200 µL system for 30 min at 4 °C (room temperature or 37 °C). After incubation, 600 µL of washing buffer was added to the incubation system and the cells were centrifuged to remove unbound aptamers. Cells were resuspended in washing buffer, filtered through a 400-mesh sieve, and analyzed via flow cytometry (BD FACS Calibur, San Jose, CA, USA) with a collection of 10,000 cells for each sample. The obtained raw data were processed with FlowJo V10 (Treestar, San Caros, CA, USA). *K*_d_ values were calculated and fitted using SigmaPlot 14.0 (Systat, Chicago, IL, USA).

### 3.4. Circular Dichroism (CD) Spectroscopy

Four oligonucleotide sequences, yly12, yly20, yly21 and yly20-7, were dissolved in PBS (containing 150 mM Na+ and 5 mM K^+^) to a final concentration of 5.0 μM. The samples were denatured via heating and stabilized at room temperature for 2 h. CD spectra were collected on a circular dichroism spectrometer (JASCO, Tokyo, Japan) with a wavelength range of 200–350 nm, a scanning speed of 500 nm/min, and a response time of 0.5 s. The optical path of the cuvette was 10 mm. The average value was taken from three scans.

CD melting was performed at a 1 °C/min ramp rate in the temperature range of 25–80 °C. The buffer baseline was subtracted before obtaining the sample spectrum. The CD values at 280 nm were normalized, and the normalized CD values at different temperatures were fitted to obtain the melting curves. *T_m_* (melting temperature) was calculated using the first derivative method with the assistance of the software Origin 2021 (Origin Lab Corp., Northampton, MA, USA).

### 3.5. Serum Stability of Aptamers

FBS (fetal bovine serum) was diluted with 1640 medium to two different concentrations (10% and 90%) and a final concentration of 1 μM of aptamers was added. The aptamer–FBS mixtures were incubated at 37 °C, and 20 µL samples were taken out at the time points of 0, 10, 20, 30, 60, 120, and 180 min, respectively. Each sample was inactivated via adding 1 µL of 5 mM EDTA and heating at 95 °C for 10 min and stored at −20 °C. Finally, all samples were mixed with DNA loading buffer (7 M urea) and denatured at 95 °C for 10 min. Samples were separated using 20% polyacrylamide denaturing (7 M urea) gel electrophoresis and then scanned and imaged with a multifunctional laser scanning imager (Cytiva Amersham Typhoon RGB, Cytiva, Marlborough, MA, USA).

### 3.6. Dimethyl Sulfate (DMS) Footprinting Assay

DMS footprinting experiments were carried out according to the procedure described previously [34]. In the 100 µL solution, 100 nM 3′ end FAM-labeled yly20 was preincubated with 300 nM L1CAM in a binding buffer for 60 min at 4 °C. 100 nM 3′ end FAM-labeled yly20 dissolved in water and PBS were used as controls. All samples were mixed with 4 μL of 10% (*v*/*v*) dimethyl sulfate in ethanol and reacted for 2 min at room temperature to methylate guanine on the DNA of yly20. The reaction was terminated through the addition of 100 µL of methylation reaction termination solution (0.6 M NaOAc (pH 8.0), 0.1 M β-mercaptoethanol, 20 μg herring sperm DNA) to the system. DNA precipitation products were obtained and dried using the phenol–chloroform extraction and ethanol precipitation method. Next. 100 μL of 20% piperidine aqueous solution was added to re-solubilize the DNA, and the system was heated at 90 °C for 30 min to react. Phenol–chloroform extraction and ethanol precipitation were repeated. The precipitated product was dissolved with 20 μL of 80% (*v*/*v*) deionized formamide aqueous solution and 4 μL of DNA electrophoresis loading buffer, and then the obtained DNA was denatured via heating at 95 °C for 5 min. Samples were added to the upper wells of a 20% urea denatured polyacrylamide gel and electrophoresed for 3 h under a 14 mA current. After electrophoresis, the gel was scanned and imaged using a multifunctional laser-scanning imager (Cytiva Amersham Typhoon RGB).

### 3.7. Plasmids and Transfection

The L1CAM full-length expression plasmid was purchased from Sino Biological, Inc. (Beijing, China). The remaining five L1CAM truncation or mutation plasmids were constructed by Beijing SBS Genetech Co., Ltd. (Beijing, China) based on the full-length plasmid. After amplification, all the plasmids were sequenced to confirm correctness by Sangon Biotech Co. Ltd. (Shanghai, China). All L1CAM plasmid transient transfection experiments were performed according to the Lipofectamine^®^ 3000 Reagent Protocol. PC3 cells were inoculated in 6-well plates and transfected with L1CAM plasmids at 70–90% confluence. The binding ability of transfected cells with different plasmids to yly20 was analyzed via flow cytometry two days after transfection. Whole proteins of the transfected cells were also extracted and analyzed via Western blotting assay for protein expression.

### 3.8. Peptide Competition Assay

The RGD (Arg-Gly-Asp) peptide and L1CAM-Ig6-25 peptide were dissolved in ddH_2_O as a stock solution (1 mM). LoVo cells were pre-incubated with 20 µM peptide for 30 min and then incubated with 200 nM FAM-labeled aptamer yly20 for 30 min. The binding of yly20 to LoVo cells with or without peptide competition was measured via flow cytometry assay, as described previously. Data were collected using a flow cytometer (BD FACS Calibur, San Jose, CA, USA) and analyzed using FlowJo V10 software (Treestar, San Caros, CA, USA).

### 3.9. Chemical Footprinting of L1CAM via HPG-Modification of Arginine

A chemical footprinting experiment of L1CAM was performed according to the method developed by Amit Ketkar et al. [36]. First, 5 μΜ L1CAM protein was preincubated with 20 μΜ yly20 or ctr-apt (control sequence) for 30 min at 4 °C, with a blank control of L1CAM samples spiked with buffer only. Then, 5 mM HPG was added to the incubation system and further incubated at room temperature in the dark for 1 h to allow HPG modification on the arginine residue. After HPG modification, 800 mM arginine was added to terminate the reaction. The samples were separated via SDS-PAGE gel electrophoresis after adding SDS loading buffer and denaturing at 95 °C. The gel was silver-stained and the appropriate protein bands were excised. The gel strips were desilvered and digested in gel with trypsin. Finally, peptide samples were collected and subjected to high-resolution LC-MS and MS/MS analysis on an Orbitrap Fusion mass spectrometer (Thermo Fisher Scientific, San Jose, CA, USA). The raw mass spectrometry data were searched using Proteome Discoverer 2.4 software (Thermo Fisher Scientific, San Jose, CA, USA) from the UniProt database to identify unmodified and HPG-modified peptides. The peak areas of the corresponding peptides were calculated using XCalibur 4.2 software (Thermo Fisher Scientific, San Jose, CA, USA) and normalized to the degree of HPG modification.

### 3.10. Molecular Modeling and Docking

The 3D structure of the Ig6 domain of L1CAM was predicted using Alphafold in the Uniport database (https://www.uniprot.org/uniprotkb/P32004, accessed on 29 June 2022). The structure of yly20 that matched the experimental results was chosen from the secondary structures predicted via the Mfold [32] website and used as a basis to model and visualize the 3D structure of yly20 via the 3dRNA/DNA website [38] (http://biophy.hust.edu.cn/new/3dRNA/create accessed on 1 July 2022). The docking of the L1CAM-Ig6 domain and the aptamer yly20 was performed by using the HDOCK (http://hdock.phys.hust.edu.cn/ accessed on 15 July 2022) tool [39] according to the prompts in the HDOCK website. Because of the larger molecular weight of yly20 than that of the Ig6 domain, yly20 was set as the receptor and the Ig6 domain was set as the ligand. Without setting the binding site information, global docking was performed directly to predict the binding complex information between the two molecules. Docking produced thousands of results, of which the top 10 ranked docking models are shown in Table S2. The docking scores were calculated using a function of ITScorePR, and a more negative docking score may imply a more possible binding model. Among the top 10 models, model 9 was the most superior binding model that matched the experimental results well.

## 4. Conclusions

In summary, through a series of experiments including nucleotide mutation, CD spectrum analysis, DMS footprinting, domain deletion mutation, amino acid mutation, peptide competition, HPG chemical footprinting, and molecular docking, the molecular mechanism of the interaction of a DNA aptamer and L1CAM was explored. Two optimized aptamers, yly20 and yly21, possessed much higher affinity (10–24 fold) to L1CAM than that of the original aptamer, yly12, at 25 and 37 °C. The aptamer yly21 showed much higher stability in serum. The optimized aptamers adopted a hairpin structure with two loops and two stems. The direct binding region to L1CAM was located in loop I and its adjacent area. Stem I mainly played the role of maintaining the binding structure. The critical nucleotides for aptamer binding were determined. The extracellular region of L1CAM contains six Ig-like domains and five fibronectin type III repeats, and the Ig6 domain was demonstrated to bind to L1CAM directly. The Ig6 domain mainly interacts with integrins that play important roles in regulating cell proliferation, differentiation, apoptosis, and migration. Therefore, the clarification of Ig6 as the binding site provides more accurate information about the molecular target for aptamer-based drug design and functional regulation and detection of L1CAM: for example, constructing multivalent ligands with molecules that bind other domains, blocking the interaction of L1CAM and integrins, and simultaneously capturing and detecting L1CAM with aptamers and antibodies that bind other domains. On the other hand, the elucidation of the binding structure and the molecular recognition mechanism of yly-series aptamers provides guidance for the design of aptamer-based targeted drugs and detection probes against L1CAM.

## Figures and Tables

**Table 1:** The apparent equilibrium dissociation constants (*K*_d_, nM) of different aptamer sequences under different temperatures.

Aptamer	4 °C	25 °C	37 °C
yly12	3.5 ± 2.4	21.6 ± 2.0	110.8 ± 21.7
yly20	2.3 ± 0.4	1.9 ± 0.3	7.6 ± 1.0
yly21	1.3 ± 0.4	0.9 ± 0.3	5.1 ± 0.5
yly31	26.5 ± 6.3	80.6 ± 15.0	876 ± 380
yly32	14.6 ± 4.6	40.8 ± 11.5	151 ± 162
yly33	6.2 ± 0.4	12.0 ± 1.1	41.4 ± 7.3
yly34	3.4 ± 0.2	7.2 ± 1.0	18.7 ± 1.6
yly20re	4.2 ± 0.5	4.4 ± 0.3	13.0 ± 9.0
yly20re2	3.2 ± 0.2	58.4 ± 9.7	98.6 ± 23.6
yly13	369 ± 349		
yly14	92 ± 64		
yly16	330 ± 298		
yly20-5-T	112 ± 20		
yly21-9-T	107 ± 28		

**Table 2:** The apparent equilibrium dissociation constant (*K*_d_, nM) of mutation sequences of yly20 under 4 °C.

Aptamer Name	*K*_d_ (4 °C)
yly20-G8A	18.9 ± 2.3
yly20-G35A	53.1 ± 9.1
yly20-C46A	46.4 ± 26.9
yly20-T45C	66.9 ± 27.8
yly20-G22A	1.3 ± 0.1
yly20-T25A	1.7 ± 0.2
yly20-C17A	3.4 ± 0.5
yly20-T16C	44.2 ± 27.8
yly20-T28C	5.3 ± 0.6
yly20-T29C	4.2 ± 0.6
yly20-T33C	9.5 ± 0.8
yly20-T1629C	155.4 ± 134.3
yly20-T2829C	55.9 ± 9.5

## Data Availability

Data is contained within the article or Supplementary Materials.

## Supplementary Materials

The following supporting information can be downloaded at: https://www.mdpi.com/article/10.3390/ijms24108612/s1.
