# Supplementary material for: Structural Optimization and Interaction Study of a DNA Aptamer to L1 Cell Adhesion Molecule
Source: Int J Mol Sci. 2023 May 11;24(10):8612. doi: 10.3390/ijms24108612 (PMC10218236; doi:10.3390/ijms24108612)
Supplement: Supplementary file 1 [file ijms-24-08612-s001.zip › ijms-2367251-supplementary.pdf]

Supporting information

Structural Optimization and Interaction Study of a DNA Aptamer to L1 Cell Adhesion Molecule

Zhenhao Long [1,2,†], Tao Bing [1,3,†], Xiangru Zhang [1,2], Jing Sheng [1,2], Shuang Zu [1,4], Weiwei Li [3,4], Xiangjun Liu [1,2], Nan Zhang [1,*] and Dihua Shangguan [1,2,4,*]

1 Beijing National Laboratory for Molecular Sciences, Key Laboratory of Analytical Chemistry for Living Biosystems, CAS Research and Education Center for Excellence in Molecular Sciences, Institute of Chemistry, Chinese Academy of Sciences, Beijing 100190, China
2 School of Chemical Sciences, University of Chinese Academy of Sciences, Beijing 100049, China
3 Zhejiang Cancer Hospital, The Key Laboratory of Zhejiang Province for Aptamers and Theranostics, Hangzhou Institute of Medicine (HIM), Chinese Academy of Sciences, Hangzhou 310022, China
4 School of Molecular Medicine, Hangzhou Institute for Advanced Study, University of Chinese Academy of Sciences, Hangzhou 310013, China
* Correspondence: hszhang@iccas.ac.cn (N.Z.); sgdh@iccas.ac.cn (D.S.)
† These authors contributed equally to this work.

Table S1. Oligonucleotides appeared in this paper.

Name	Sequences (5’ to 3’)
yly12	AGGATAGGGGGTAGCTCGGTCGTGTTTTTGGGTTGTTTGGTGGGTCTTCTG
yly13	AGGATAGGGGGTAGCTCGGTCGTGTTTTTGGGTTGTTTGGTGGGTCTT
yly14	ATAGGGGGTAGCTCGGTCGTGTTTTTGGGTTGTTTGGTGGGTCTTCTG
yly16	TAGGGGGTAGCTCGGTCGTGTTTTTGGGTTGTTTGGTGGGTCTTCTG
yly20	CAGATAGGGGGTAGCTCGGTCGTGTTTTTGGGTTGTTTGGTGGGTCTTCTG
yly21	AGCGCAGATAGGGGGTAGCTCGGTCGTGTTTTTGGGTTGTTTGGTGGGTCTTCTGCGCT
yly20re	TTTGGGTTGTTTGGTGGGTCTTCTGCAGATAGGGGGTAGCTCGGTCGTGTT
yly20re2	TTTGGGTTGTTTGGTGGGTCTTCTGTTTCAGATAGGGGGTAGCTCGGTCGTGTT
yly20-5-T	CAGAAGGGGGTAGCTCGGTCGTGTTTTTGGGTTGTTTGGTGGGTCTTCTG
yly21-9-T	AGCGCAGAAGGGGGTAGCTCGGTCGTGTTTTTGGGTTGTTTGGTGGGTCTTCTGCGCT
yly31	GGTATAGGGGGTAGCTCGGTCGTGTTTTTGGGTTGTTTGGTGGGTCTTCTG
yly32	GGAATAGGGGGTAGCTCGGTCGTGTTTTTGGGTTGTTTGGTGGGTCTTCTG
yly33	GGGATAGGGGGTAGCTCGGTCGTGTTTTTGGGTTGTTTGGTGGGTCTTCTG
yly34	CGGATAGGGGGTAGCTCGGTCGTGTTTTTGGGTTGTTTGGTGGGTCTTCTG
yly20-G7A	CAGATAAGGGGTAGCTCGGTCGTGTTTTTGGGTTGTTTGGTGGGTCTTCTG
yly20-G8A	CAGATAGAGGGTAGCTCGGTCGTGTTTTTGGGTTGTTTGGTGGGTCTTCTG
yly20-G9A	CAGATAGGAGGTAGCTCGGTCGTGTTTTTGGGTTGTTTGGTGGGTCTTCTG
yly20-G10A	CAGATAGGGAGTAGCTCGGTCGTGTTTTTGGGTTGTTTGGTGGGTCTTCTG
yly20-G11A	CAGATAGGGGATAGCTCGGTCGTGTTTTTGGGTTGTTTGGTGGGTCTTCTG
yly20-G14A	CAGATAGGGGGTAACTCGGTCGTGTTTTTGGGTTGTTTGGTGGGTCTTCTG
yly20-C15A	CAGATAGGGGGTAGATCGGTCGTGTTTTTGGGTTGTTTGGTGGGTCTTCTG
yly20-T16C	CAGATAGGGGGTAGCCCGGTCGTGTTTTTGGGTTGTTTGGTGGGTCTTCTG
yly20-C17A	CAGATAGGGGGTAGCTAGGTCGTGTTTTTGGGTTGTTTGGTGGGTCTTCTG
yly20-C1517A	CAGATAGGGGGTAGATAGGTCGTGTTTTTGGGTTGTTTGGTGGGTCTTCTG
yly20-G18A	CAGATAGGGGGTAGCTCAGTCGTGTTTTTGGGTTGTTTGGTGGGTCTTCTG
yly20-G1418A	CAGATAGGGGGTAACTCAGTCGTGTTTTTGGGTTGTTTGGTGGGTCTTCTG
yly20-G22A	CAGATAGGGGGTAGCTCGGTCATGTTTTTGGGTTGTTTGGTGGGTCTTCTG
yly20-T25A	CAGATAGGGGGTAGCTCGGTCGTGATTTTGGGTTGTTTGGTGGGTCTTCTG
yly20-T28C	CAGATAGGGGGTAGCTCGGTCGTGTTTCTGGGTTGTTTGGTGGGTCTTCTG
yly20-T29C	CAGATAGGGGGTAGCTCGGTCGTGTTTTCGGGTTGTTTGGTGGGTCTTCTG
yly20-G31A	CAGATAGGGGGTAGCTCGGTCGTGTTTTTGAGTTGTTTGGTGGGTCTTCTG
yly20-T33C	CAGATAGGGGGTAGCTCGGTCGTGTTTTTGGGCTGTTTGGTGGGTCTTCTG
yly20-T1629C	CAGATAGGGGGTAGCCCGGTCGTGTTTTCGGGTTGTTTGGTGGGTCTTCTG
yly20-T162933C	CAGATAGGGGGTAGCCCGGTCGTGTTTTCGGGCTGTTTGGTGGGTCTTCTG
yly20-G35A	CAGATAGGGGGTAGCTCGGTCGTGTTTTTGGGTTATTTGGTGGGTCTTCTG
yly20-G40A	CAGATAGGGGGTAGCTCGGTCGTGTTTTTGGGTTGTTTGATGGGTCTTCTG
yly20-G43A	CAGATAGGGGGTAGCTCGGTCGTGTTTTTGGGTTGTTTGGTGAGTCTTCTG
yly20-C46A	CAGATAGGGGGTAGCTCGGTCGTGTTTTTGGGTTGTTTGGTGGGTATTCTG
yly20-7	TAGGGGGTAGCTCGGTCGTGTTTTTGGGTTGTTTGGTGGGTCT
Ctr-sq	CAGATAGGAGGTAGCTCGGTCGTGTTTTTGGGTTGTTTGGTGGGTCTTCTG

Red letters indicate the mutated nucleotides.

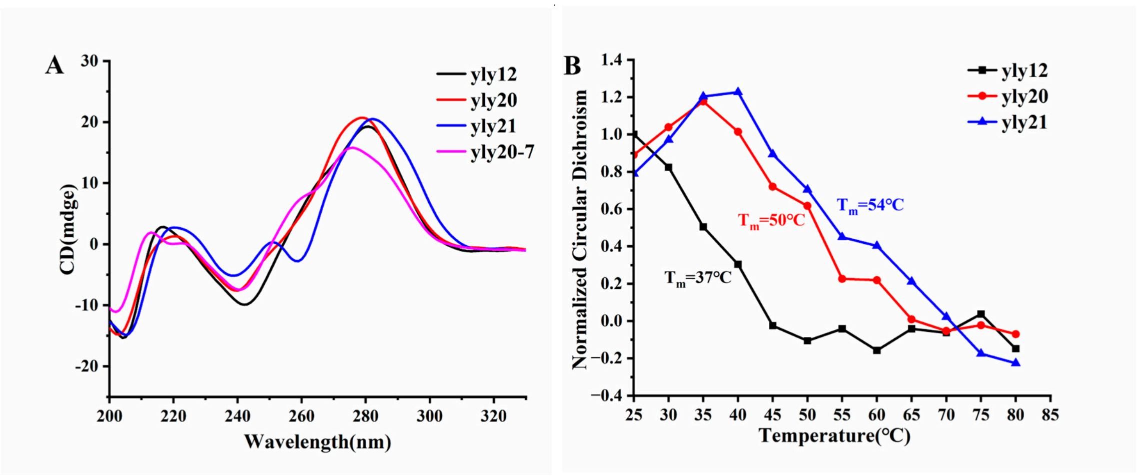

Figure S1. (A) The CD spectra of yly12, yly20, yly21 and yly20-7. (B) CD melting curves of yly12, yly20 and yly21. The concentration of DNA was 5 μM in PBS (150 mM Na^+ and 5mM K^+). The optical path length of the cell was 10 mm.

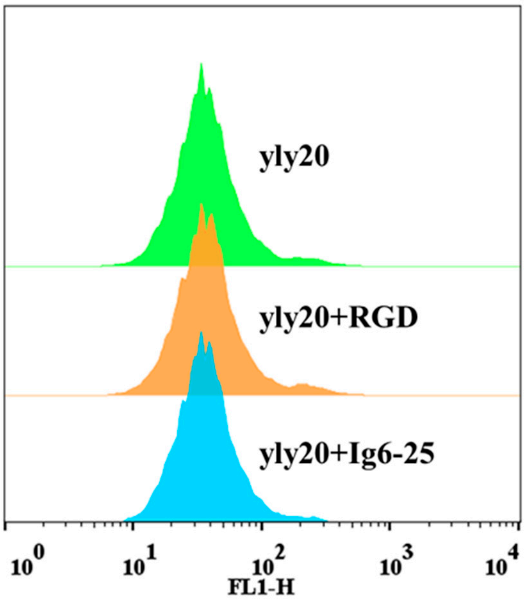

Figure S2. The binding of yly20 under the competition of RGD and Ig6-25 peptides.

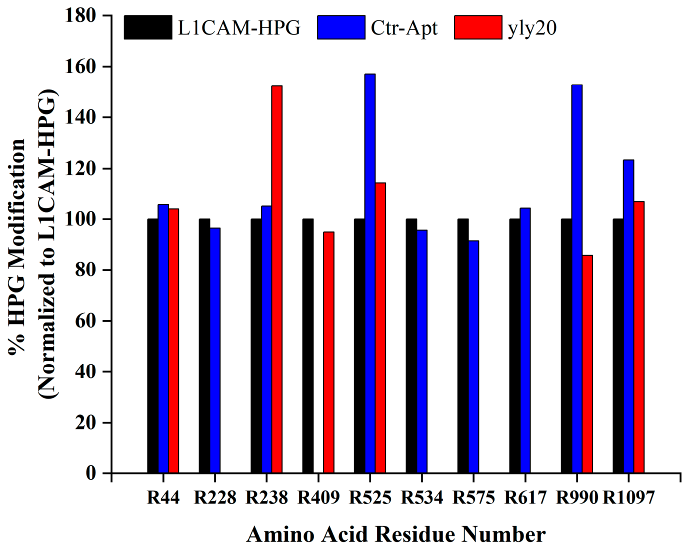

Figure S3. LC-MS analysis of HPG-modified arginine sites. The fraction of 10 detected HPG-modified arginines to total arginines.

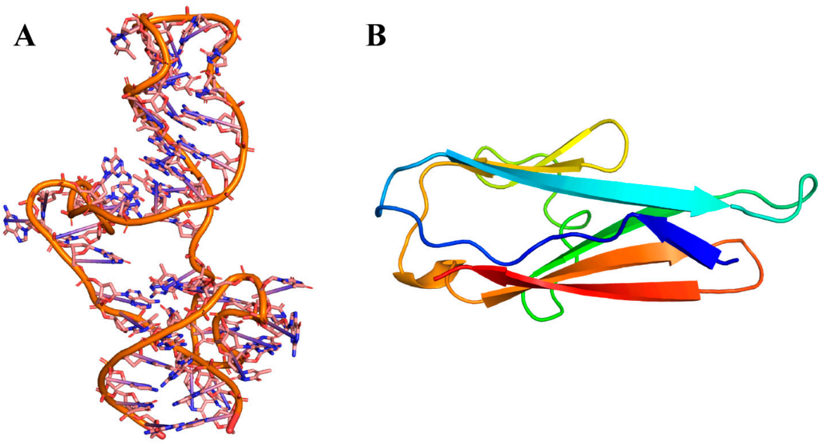

Figure S4. The 3D structure of yly20 (A) and Ig6 domain (B).

Table S2. The top10 results of yly20-Ig6 domain docking models.

Rank	1	2	3	4	5	6	7	8	9	10
Docking Score	-167.36	-178	-176.35	-165.31	-150.47	-148.4	-142.86	-130.21	-182.12	-165.54
Ligand RMSD	49.7	54.21	35.69	49.27	40.02	47.56	40.7	45.88	39.23	42.42

a Row 1: The ranks of the models.

b Row 2: The docking energy scores (unit: kcal/mol).

c Row 3: The ligand RMSDs from the input structures or modeled structures by homology modeling (unit: Å).

Table S3. The residues of aptamer yly20 and L1CAM Ig6 in interactions obtained by molecular docking.

yly20 residues	L1CAM Ig6 residues	Distance (Å)
G7	S594	2.98
G7	S550	3.19
G8	T552	2.22
G8	R554	2.94
G8	V592	3.07
G8	V599	2.84
G9	R554	2.07
G10	R554	4.08
G10	E601	3.41
G11	G557	3.59
G11	R558	4.44
T37	Q561	2.58
T37	G564	2.23
T37	D565	2.61
T38	I571	3.16
G39	P549	2.15
G39	S550	3.7
G39	D573	3.18
G39	G574	2.91
G40	P549	4.82
G42	P545	2.5
G42	S546	3.44
G43	S546	3.67
G43	Q548	4.55
G44	Q548	3.41
T45	E596	3.68
T45	Q548	3.73
C46	E596	3.27
T47	L597	4.33

Table S4. L1CAM-derived peptides (trypsin-digested) identified by mass spectrometry.

Peptide Sequence (Trypsin cleavage sites in parentheses)	m/z	M.W.	Charge State	Residue (Start-End)
(R)rLVVFPTDDISLK(C)	817.44	1634.88	2	044-056
(R)RLVVFPTDDISLK(C)	751.43	1502.86	2	044-056
(R)LVVFPTDDISLK(C)	673.38	1346.76	2	044-056
(K)CEASGKPEVQFR(W)	675.32	1350.65	2	057-068
(R)WTRDGVHFKPK(E)	456.91	1370.74	3	069-079
(R)DGVHFKPK(E)	463.75	927.51	2	072-079
(R)DGVHFKPKEELGVTVYQSPHSGSFTITGNNSNFAQR(F)	987.24	3948.95	4	072-107
(K)EELGVTVYQSPHSGSFTITGNNSNFAQR(F)	1013.48	3040.45	3	080-107
(R)FQGIYR(C)	391.71	783.42	2	108-113
(K)LGTAMSHEIR(L)	371.52	1114.57	3	120-129
(K)LGTAmSHEIR(L)	376.85	1130.56	3	120-129
(R)LMAEGAPK(W)	408.21	816.43	2	130-137
(R)LmAEGAPK(W)	416.21	832.42	2	130-137
(R)LmAEGAPKWPK(W)	414.55	1243.66	3	130-140
(R)LMAEGAPKWPK(W)	409.22	1227.66	3	130-140
(R)IYWmNSK(I)	478.73	957.45	2	169-175
(R)IYWMNSK(I)	470.73	941.46	2	169-175
(K)ILHIKQDER(V)	383.88	1151.65	3	176-184
(R)TIIQKEPIDLR(V)	662.89	1325.78	2	218-228
(R)TIIQKEPIDLRVK(A)	388.24	1552.94	4	218-230
(R)TIIQKEPIDLrVK(A)	561.66	1684.97	3	218-230
(K)EPIDLR(V)	371.21	742.41	2	223-228
(K)EPIDLrVK(A)	550.80	1101.60	2	223-230
(R)VKATNSMIDR(K)	567.30	1134.60	2	229-239
(R)VKATNSmIDR(K)	575.30	1150.59	2	229-239
(R)VKATNSmIDRKPR(L)	382.96	1531.84	4	229-241
(K)ATNSMIDR(K)	453.72	907.43	2	231-238
(K)ATNSmIDR(K)	461.71	923.43	2	231-238
(K)ATNSmIDRKPR(L)	434.89	1304.68	3	231-241
(K)ATNSmIDrKPR(L)	478.90	1436.70	3	231-241
(K)WLRPSGPMPADR(V)	460.90	1382.70	3	276-287
(K)WLRPSGPmPADR(V)	466.23	1398.70	3	276-287
(K)TLQLLK(V)	357.74	715.47	2	296-301
(K)TLQLLKVGEEDDGEYR(C)	932.47	1864.94	2	296-311
(R)VGEEDDGEYR(K)	584.24	1168.48	2	302-311
(R)VGEEDDGEYRCLAENSLGSAR(H)	756.67	2270.01	3	302-322
(R)CLAENSLGSAR(H)	560.27	1120.54	2	312-322
(R)HAYYVTVEAAPYWLHKPQSHLYGPGETAR(L)	835.41	3341.66	4	323-351
(R)LDCQVQGRPQPEVTWR(I)	637.32	1911.95	3	352-367
(R)INGIPVEELAK(D)	591.34	1182.68	2	368-378
(R)INGIPVEELAKDQK(Y)	517.95	1553.86	3	368-381
(R)INGIPVEELAKDQKYR(I)	624.34	1873.02	3	368-383
(R)GALILSNVQPSDTMVTQCEAR(N)	744.37	2233.10	3	387-407
(R)NRHGLLLANAYIYVVQLPAK(I)	751.10	2253.29	3	408-428
(R)NrHGLLLANAYIYVVQLPAK(I)	800.77	2402.30	3	408-428
(R)HGLLLANAYIYVVQLPAK(I)	991.57	1983.14	2	410-427
(K)AFGAPVPSVQWLDEDGTTVLQDER(F)	876.76	2630.27	3	450-473
(R)FFPYANGTLGIR(D)	677.86	1355.72	2	474-485
(K)VKDATQITQGPR(S)	437.91	1313.72	3	514-525
(K)VKDATQITQGPrSTIEK(K)	668.01	2004.04	3	514-530

(K)VKDATQITQGPRSTIEK(K)	468.01	1872.02	4	514-530
(K)DATQITQGPR(S)	543.28	1086.56	2	516-525
(R)STIEKK(G)	352.71	705.41	2	526-531
(K)GSRVTFTCQASFDPSLQPSITWR(G)	880.44	2641.31	3	532-554
(K)GSrVTFTCQASFDPSLQPSITWR(G)	930.10	2790.31	3	532-554
(R)VTFTCQASFDPSLQPSITWR(G)	761.37	2284.11	3	535-555
(R)GDGRDLQELGDSDK(Y)	501.56	1504.69	3	555-568
(R)GDGRDLQELGDSDKYFIEDGR(L)	795.03	2385.10	3	555-575
(R)DLQELGDSDK(Y)	559.76	1119.52	2	559-568
(R)DLQELGDSDKYFIEDGR(L)	666.64	1999.93	3	559-575
(K)YFIEDGR(L)	449.71	899.43	2	569-575
(K)YFIEDGrLVIHSLDYSDQGNYSCVASTELD VVESR(A)	1066.48	4265.90	4	569-603
(R)AQLLVVGSPGPVPR(L)	463.27	1389.82	3	604-617
(R)AQLLVVGSPGPVPrLVLSDLHLLTQSQVR(V)	1074.94	3224.83	3	604-642
(R)AQLLVVGSPGPVPRLVLSDLHLLTQSQVR(V)	1030.93	3092.80	3	604-642
(R)LVLSDLHLLTQSQVR(V)	861.00	1722.00	2	618-632
(R)LVLSDLHLLTQSQVRVSWSPAEDHNAPIEK(Y)	676.56	3382.78	5	618-647
(R)VSWSPAEDHNAPIEK(Y)	559.94	1679.81	3	633-647
(R)VSWSPAEDHNAPIEKYDIEFEDK(E)	906.42	2719.26	3	633-655
(P)VSWSPAEDHNAPIEKYDIEFEDKEmAPEK(W)	1140.19	3420.57	3	633-672
(P)VSWSPAEDHNAPIEKYDIEFEDKEMAPEK(W)	851.14	3404.56	4	633-672
(K)YDIEFEDK(E)	529.24	1058.47	2	648-655
(K)YDIEFEDKEmAPEK(W)	586.59	1759.78	3	648-661
(K)YDIEFEDKEMAPEK(W)	871.89	1743.78	2	648-661
(K)YDIEFEDKEMAPEKWYSLGK(V)	826.05	2478.16	3	648-667
(K)YDIEFEDKEmAPEKWYSLGK(V)	831.38	2494.15	3	648-667
(K)WYSLGK(V)	376.70	753.39	2	662-667
(K)LSPYVHYTFR(V)	427.55	1282.66	3	679-688
(K)LSPYVHYTFRVTAINKYGPGEPSPVSETVVTP EAAPEK(N)	1032.78	4131.12	4	679-716
(K)LSPYVHYTFRVTAINK(Y)	636.34	1909.03	3	679-694
(R)VTAINK(Y)	322.70	645.40	2	689-694
(R)VTAINKYGPGEPSPVSETVVTPEAAPEK(N)	955.83	2867.48	3	689-716
(R)VTAINKYGPGEPSPVSETVVTPEAAPEKNPV DVK(G)	879.96	3519.82	4	689-722
(K)YGPGEPSPVSETVVTPEAAPEK(N)	1120.55	2241.10	2	695-716
(K)YGPGEPSPVSETVVTPEAAPEKNPVDVK(G)	964.48	2893.45	3	695-722
(K)YGPGEPSPVSETVVTPEAAPEKNPVDVKGEG NETTNMVITWKPLR(M)	964.08	4820.41	5	695-739
(K)GEGNETTNmVITWKPLR(W)	653.99	1961.98	3	723-739
(K)GEGNETTNMVITWKPLR(W)	648.66	1945.97	3	723-739
(R)WMDWNAPQVQYR(V)	796.87	1593.73	2	740-751
(R)WmDWNAPQVQYR(V)	804.86	1609.73	2	740-751
(R)VQWRPQGTR(G)	375.87	1127.61	3	752-759
(R)GPWQEQIVSDPFLVVSNTSTFVPYEIK(V)	1026.86	3080.57	3	761-787
(K)VQAVNSQGK(G)	465.25	930.50	2	788-796
(K)GPEPQVTIGYSGEDYPQAIPELEGIEILNSS AVLVK(W)	1270.98	3812.95	3	797-832
(K)WRPVDLAQVK(G)	403.90	1211.69	3	833-842
(R)GYNVTYWR(E)	529.25	1058.51	2	847-854
(R)WQPPLSHNGVLTGYVLSYHPLDEGGK(G)	954.81	2864.44	3	938-963
(R)WQPPLSHNGVLTGYVLSYHPLDEGGKGQ LSFNLR(D)	944.98	3779.94	4	938-971
(R)WQPPLSHNGVLTGYVLSYHPLDEGGKGQ LSFNLRDPELR(T)	731.70	4390.23	6	938-976
(K)GQLSFNLR(D)	467.26	934.51	2	964-971

(K)GQLSFNLRDPELR(T)	514.94	1544.83	3	964-976
(R)THNLTDLSPHLR(Y)	467.92	1403.75	3	977-988
(R)YRFQLQATTK(E)	418.56	1255.68	3	989-998
(R)YrFQLQATTK(E)	693.85	1387.70	2	989-998
(R)FQLQATTKEGPGEAIVR(E)	615.00	1844.99	3	991-1007
(R)FQLQATTK(E)	468.26	936.52	2	991-998
(K)EGPGEAIVR(E)	463.75	927.49	2	999-1007
(R)EGGTMALSGISDFGNISATAGENYSVVS WVPK(E)	1081.52	3244.56	3	1008-1039
(R)EGGTmALSGISDFGNISATAGENYSVVS WVPK(E)	1086.86	3260.57	3	1008-1039
(R)FHILFK(A)	402.24	804.48	2	1047-1052
(R)FHILFKALGEEK(G)	477.27	1431.80	3	1047-1058
(K)ALGEEKGGASLSPQYVSYNQSSYTQWD LQPDTDYEIHLFK(E)	1141.29	4565.17	4	1052-1192
(K)ALGEEK(G)	323.17	646.34	2	1053-1058
(K)GGASLSPQYVSYNQSSYTQWDLQPD TDYEIHLFK(E)	984.46	3937.83	4	1059-1192
(R)MFrHQMAVK(T)	426.54	1279.61	3	1095-1103
(R)MFRHQmAVK(T)	387.86	1163.58	3	1095-1103
(R)mFrHQmAVK(T)	655.80	1311.60	2	1095-1103
(R)mFRHQmAVK(T)	393.19	1179.58	3	1095-1103
(R)mFrHQMAVK(T)	431.87	1295.60	3	1095-1103
(R)HQMAVK(T)	356.69	713.38	2	1098-1103
(R)HQmAVK(T)	364.69	729.37	2	1098-1103

Arg residues highlighted in red lowercase ‘r’ were modified by HPG (+132) and lowercase ‘m’ indicated oxidation (+16) of Met residues.
